# A Case of Pediatric Aspiration of a Metallic Spring

**DOI:** 10.7759/cureus.9987

**Published:** 2020-08-24

**Authors:** Sarah Callaham, Andrew Kelly, Levi Stevens, David Thomas, Michele M Carr

**Affiliations:** 1 Otolaryngology - Head and Neck Surgery, West Virginia University School of Medicine, Morgantown, USA; 2 Otolaryngology, Jacobs School of Medicine and Biomedical Sciences at the University at Buffalo, Buffalo, USA; 3 Pediatrics, West Virginia University School of Medicine, Morgantown, USA

**Keywords:** aspiration, pediatric, afb, otolaryngology, rigid bronchoscopy

## Abstract

Prolonged retention of a foreign body after aspiration can lead to numerous respiratory complications. We present a case in which an unwitnessed aspiration of a metal spring by a child led to several months of unilateral wheezing and subsequent physical changes in his left mainstem bronchus. The prompt removal of an airway foreign body requires a high index of suspicion by the physician in order to facilitate proper workup to confirm the diagnosis, allow for prompt management, and minimize damage to the airway.

## Introduction

Foreign body aspiration events affect approximately 2.5 million children in the United States each year [1]. Given the wide range of clinical presentations and symptom overlap with other childhood respiratory disorders, airway foreign bodies (AFBs) are notoriously difficult to diagnose. Consequences of AFBs vary from temporary airway sequelae to catastrophic complications, such as anoxic brain injury, pulmonary hemorrhage, and death [2]. Historically reported mortality rates due to AFBs fluctuate from 1% to nearly 3% in the United States [1,3]. Some AFBs may not be clinically evident in the acute setting, and prolonged retention can lead to airway stenosis and granulation [4,5]. We present a unique case of retained AFB in a pediatric patient, along with subsequent management and airway complications.

## Case presentation

**Figure 1:**
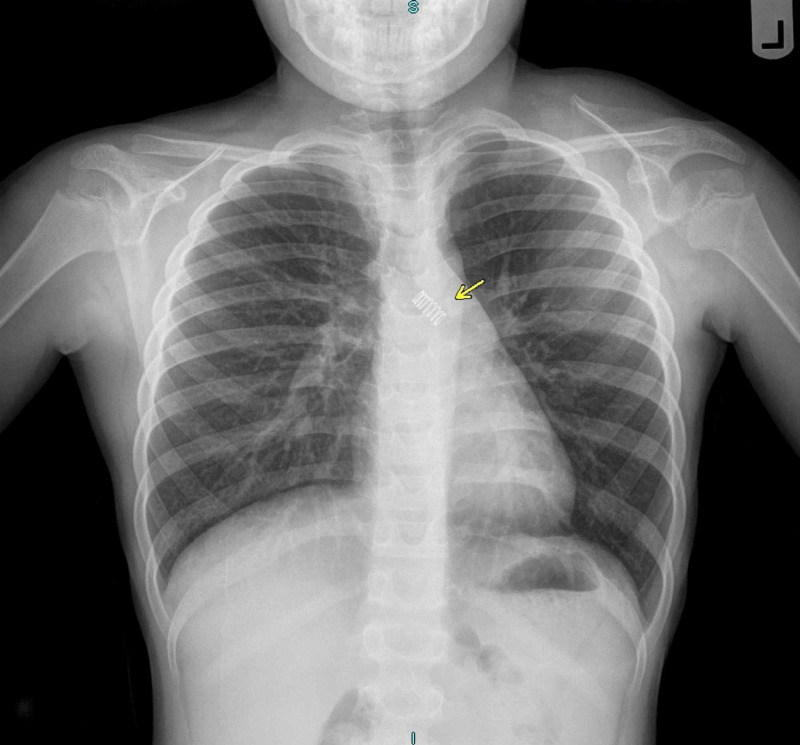
Posteroanterior (PA) chest radiograph reveals radiopaque spring in left main bronchus without associated pulmonary consolidation or atelectasis. No effusions or discrete parenchymal masses are evident.

**Figure 2:**
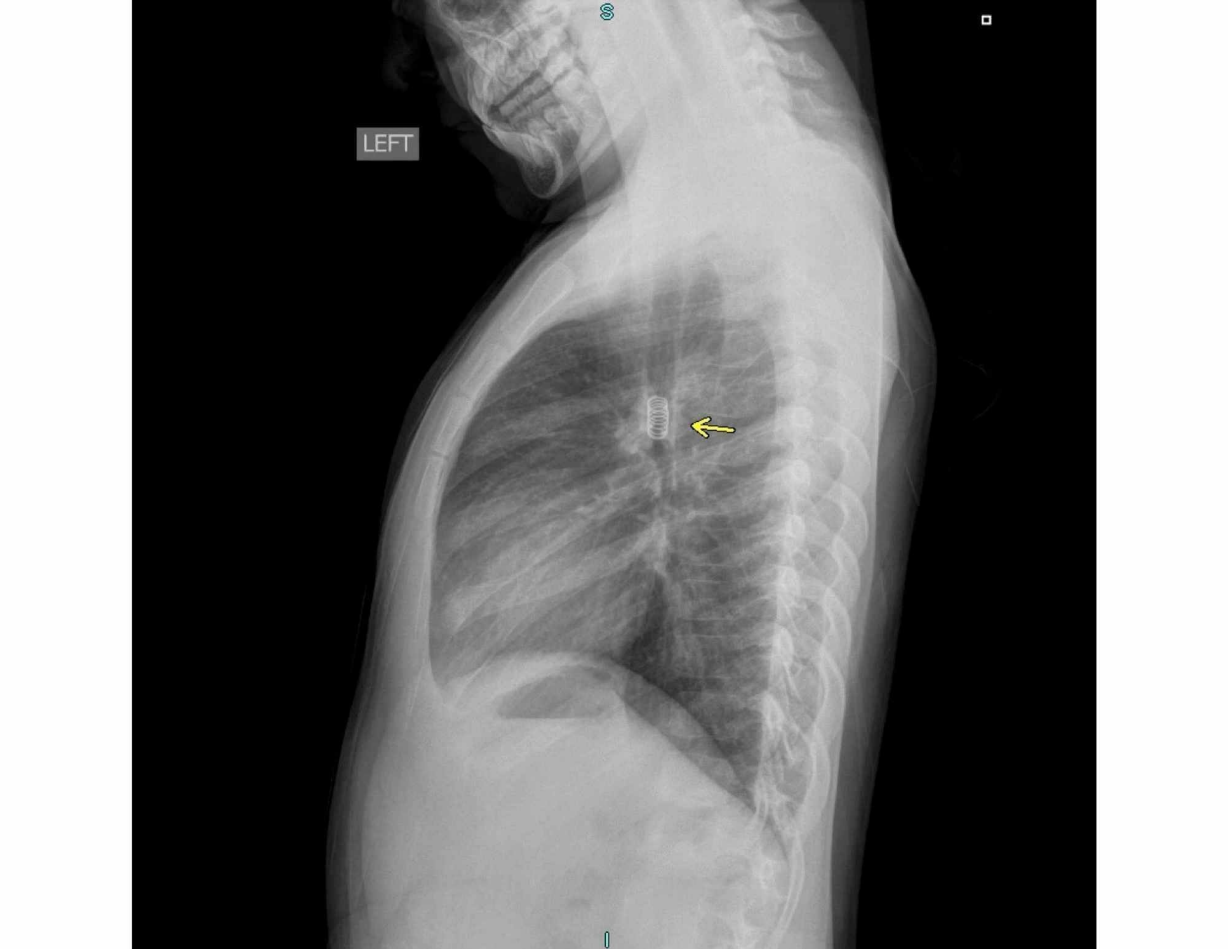
Lateral chest radiograph reveals radiopaque spring in left main bronchus without associated pulmonary consolidation or atelectasis. No effusions or discrete parenchymal masses are evident.

An eight-year-old male presented to his pediatrician with a four-month history of wheezing and dyspnea on exertion. He was prescribed antibiotics and albuterol for presumed asthma with no improvement in symptoms. Following a failed inhaled corticosteroid treatment at his second outpatient visit, his pediatrician ordered posteroanterior (PA) and lateral chest radiographs. These revealed a radiopaque spring in the left main bronchus (Figures 1, 2). The patient was transferred to the emergency department via private automobile by his mother and directly admitted to the pediatric service, after which otolaryngology was consulted. 

The patient confirmed an unwitnessed aspiration of the metallic spring from a toy approximately four months prior to admission, although he did not alert his mother or pediatrician at the time. Immediately after the aspiration event, he did not experience respiratory symptoms and was able to perform his daily activities. His only complaint for some time was difficulty breathing with physical activity. Vital signs upon arrival to the hospital were as follows: heart rate was observed to be 82 beats/minute, respiratory rate was 20 breaths/minute, oxygen saturation was 100% on room air, and temperature was 36.8°C. Physical examination revealed symmetric lung inflation and unilateral, biphasic wheezing on auscultation. Coarse breath sounds were auscultated in the left upper lobe, along with decreased movement of air at the left lung base. No tactile fremitus was noted. Neither nasal flaring nor retractions were observed. All other systems were reviewed and found to be negative or non-contributory. The patient remained calm and cooperative throughout the evaluation. Medical history was significant only for attention deficit disorder.

**Figure 3:**
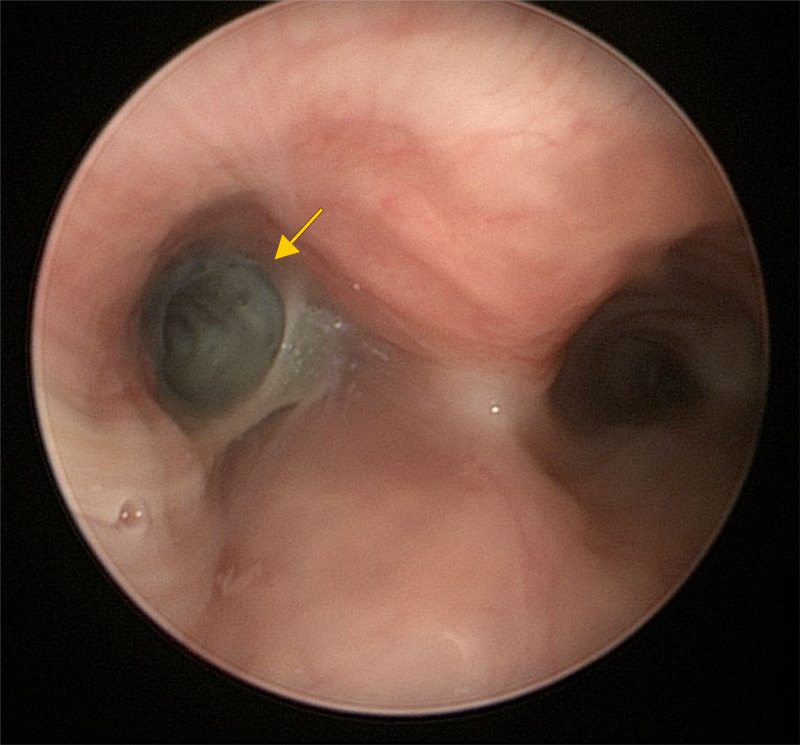
Rigid bronchoscopy with left and right main bronchi in view. Metallic spring covered in green purulent secretions seen in left main bronchus.

The patient was started on intravenous dexamethasone and inhaled nebulized budesonide with the intention of reducing expected edema related to the prolonged retention of the foreign body, along with famotidine for reflux prophylaxis. He underwent direct laryngoscopy and rigid bronchoscopy for foreign body removal on hospital day 4. Pediatric otolaryngology encountered a metallic spring coated in thick, purulent secretions in the left main bronchus (Figure 3). The spring was freely mobile and safely removed with optical alligator forceps through the bronchoscope. The extracted spring measured 1.3 cm in length and 0.6 cm in diameter. Small diffuse fragments of black material thought to be debris from the AFB were seen on the airway mucosa after extraction. Remnant mucus and small black particles were evacuated through the rigid bronchoscope using suction under direct visualization. The pediatric pulmonology team followed with a flexible bronchoscopy and left bronchoalveolar lavage (BAL). Fluid obtained during BAL appeared bloody and revealed normal cell count with differential: 68% neutrophils, 1% lymphocytes, 27% epithelial cells, and many red blood cells (RBCs). BAL fluid cell count was as follows: 300 white blood cells (WBCs) and 41,000 RBCs.

**Figure 4:**
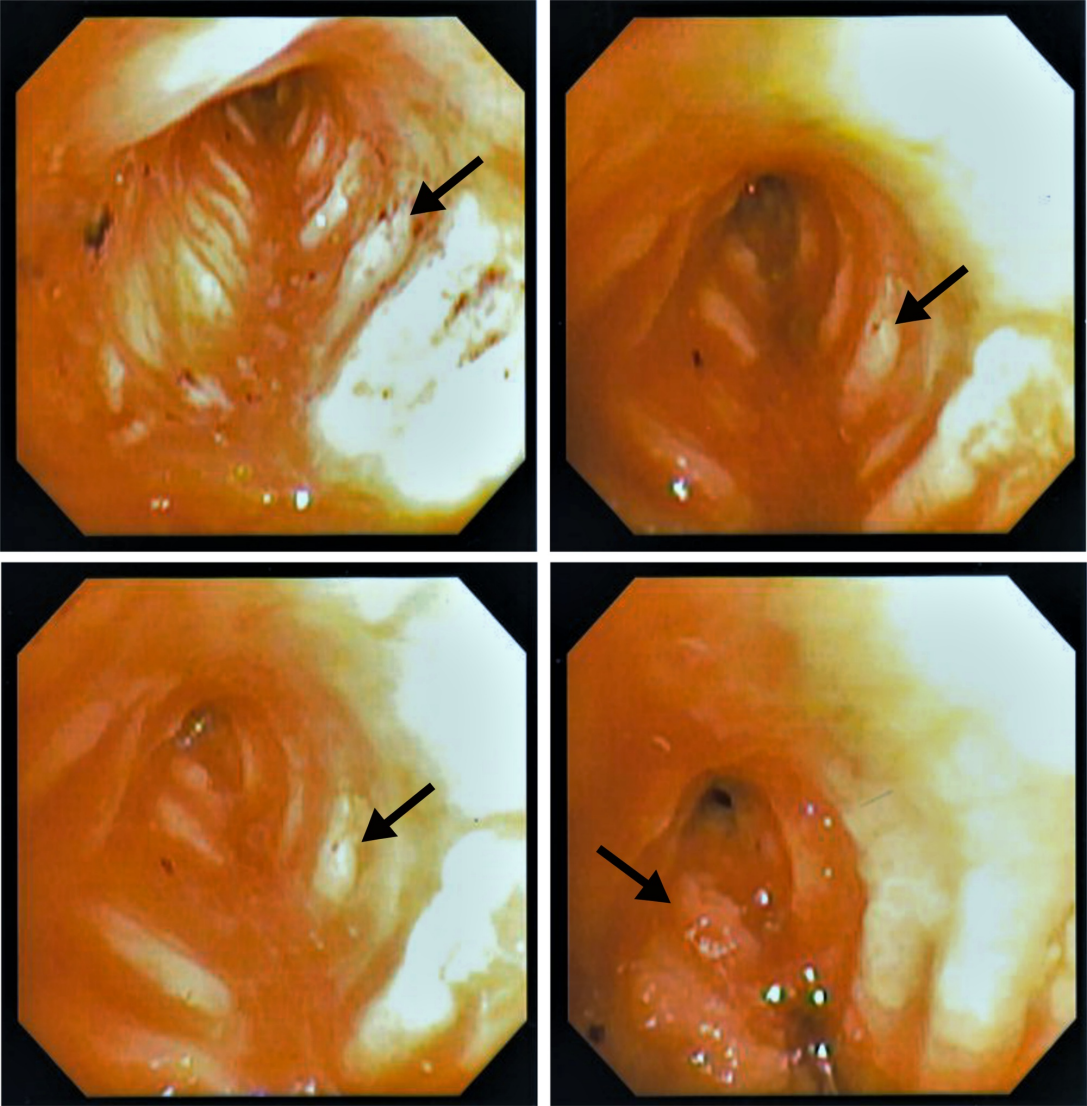
Flexible bronchoscopy of left main bronchus after spring removal. Bronchial granulation tissue has formed in ridges and stenosis is observed.

Flexible bronchoscopy revealed extensive granulation tissue and edema deep in the left main bronchus. Stenosis related to edema and granulation tissue was measured to be 1.5 mm (Figure 4). The bronchoscope was not passed through the stenotic portion to avoid irritating the granulation tissue and causing hemorrhage. 

After the spring was extracted, the patient’s biphasic wheezing resolved and his postoperative chest radiographs demonstrated normal left lung inflation. The patient was continued on IV dexamethasone until discharge on postoperative day 2. Flovent inhaler and famotidine prescriptions were given prior to discharge, and he was instructed to continue their use for six to eight weeks.

Postoperative follow-up was conducted by pediatric pulmonology. Physical examination at both one month and three months postoperative revealed no wheezes or crackles on auscultation. The patient denied any shortness of breath or exercise intolerance. Follow-up pulmonary function testing (PFTs) indicated no evidence of obstructive or restrictive lung disease. Flexible bronchoscopy performed at three months to evaluate for airway stenosis showed great improvement compared to the previous perioperative flexible bronchoscopy. Only 10% obstruction of the left main bronchus remained. The mucosa appeared normal without any inflammatory changes or postobstructive secretions. No further flexible bronchoscopies were recommended by pediatric pulmonology.

## Discussion

Foreign body aspiration is a common, potentially life-threatening condition in children. A high index of suspicion is necessary to promptly diagnose and prevent the potential sequelae of prolonged retention. Predictors of an AFB include history of witnessed choking, paroxysmal coughing, new onset of unilateral wheezing, recurrent persistent wheezing, noisy breathing, stridor, dysphonia, unilateral reduced air entry on auscultation, and abnormal chest x-ray findings [6-8]. While a history of witnessed choking is a significant indicator of AFB, choking may not always be elicited by history as the aspiration event may not be observed. Despite the presence of an AFB, many children exhibit mild or absent symptoms on examination [7]. Approximately 20% of children are treated for a different respiratory disorder before AFB is correctly diagnosed [8].

Chest radiographs enable the provider to quickly diagnose radiopaque AFBs. A negative chest radiograph cannot be used to rule out the presence of an AFB, as only 16% of aspirated objects are radiopaque [7,8]. Other chest radiograph findings in cases of AFB include unilateral atelectasis, local hyperinflation, obstructive emphysema, pulmonary infiltrates, and mediastinal shift; however, none of these findings were observed in this particular patient [8,9]. In this case, the shape of the aspirated object was a primary factor in delayed diagnosis. The hollow nature of the spring allowed the patient to maintain adequate airflow with few symptoms in the months preceding the diagnosis. It was the eventual development of recurrent, biphasic, unilateral wheezing that incited further workup for an AFB.

The nature of the foreign body not only determines the degree of airway compromise, but also the type and timing of the body’s response. Organic material typically produces more rapid mucosal inflammation and occlusion of airways, while inorganic material remains asymptomatic for longer periods of time [8,10]. Complications of a retained AFB include recurrent pneumonia, recurrent hemoptysis, bronchiectasis, bronchial strictures, and edema due to inflammation and prolonged irritation of the bronchial lining [8,10]. Additionally, intraoperative granulations were found in the majority of both adults and children with retained foreign bodies [11,12]. Granulation tissue formation and stenosis of the left main bronchus present in this case after foreign body removal were due to prolonged retention. These potentially long-term changes provide a clear example of why a high index of suspicion for AFB is warranted even when acute airway compromise is not present.

Rigid bronchoscopy is the standard of care for the definitive diagnosis of AFB and is regarded as the safest modality for their removal in pediatric patients [8,10,13]. In comparison to alternative methods like flexible bronchoscopy, rigid bronchoscopy offers greater access to subglottic airways for oxygenation and greater space to maneuver instruments [10]. A retrospective study by Goyal et al. reported that only 37% of tracheobronchial foreign bodies were successfully removed via flexible bronchoscopy. This low success rate was attributed to the narrow channel provided by flexible bronchoscopy, which limited the extraction tools available for use. Furthermore, this study found that 94% of the AFBs unsuccessfully extracted by flexible bronchoscopy were removed using rigid bronchoscopy [13]. However, flexible bronchoscopy can be useful following AFB extraction as it can allow for greater visualization of deeper structures in the airway. There has been some discussion surrounding the use of CT as an alternative to rigid bronchoscopy for definitive diagnosis of AFB [14]. While CT may provide an avenue to decrease the number of negative bronchoscopies [14], it is not normally used in order to avoid unnecessary radiation exposure in children [15]. Postoperative bronchoscopy following AFB is necessitated in two cases: suspected residual foreign body and presence of significant intraluminal granulation tissue such as in this case [12]. Additionally, postoperative bronchoscopy can facilitate the management of bronchial stenosis if necessary.

## Conclusions

We present the case of a child with unilateral biphasic wheezing who was found to have aspirated a metal spring months before its eventual discovery. Extraction was uncomplicated, and there was limited residual stenosis from prolonged airway irritation on follow-up. Clinicians should keep a high index of suspicion for AFB in the setting of unilateral wheezing, even if a clear inciting event is not apparent. Prompt management, including a chest radiograph and rigid bronchoscopy for diagnosis and removal, is essential to achieve optimal outcomes, as prolonged retention can lead to morbidity and extended medical care.
